# Editorial: The Interplay of Host-Microbiome-Pathogen on Infectious Diseases

**DOI:** 10.3389/fmicb.2022.924807

**Published:** 2022-05-18

**Authors:** John J. Maurer, Augusto Simoes-Barbosa, Timothy J. Johnson, Tyrrell Conway

**Affiliations:** ^1^Department of Animal and Poultry Sciences, Virginia Tech, Blacksburg, VA, United States; ^2^School of Biological Sciences, University of Auckland, Auckland, New Zealand; ^3^College of Veterinary Medicine, University of Minnesota, Minneapolis, MN, United States; ^4^Department of Microbiology and Molecular Genetics, Oklahoma State University, Stillwater, OK, United States

**Keywords:** microbiome, pathogen exclusion, dysbacteriosis, community diversity, probiotic

Animal microbiomes are formed by communities of microorganisms that display remarkable niche specificity. Virtually, all exposed surfaces and many deep organs (once thought to be sterile) are colonized by specific microbial consortia. When developed properly, microbiomes are known to play key roles in animal development and physiology; helping maintaining the normal functions of the host (i.e., eubiosis). On the other hand, infections are often accompanied by microbial imbalances (i.e., dysbiosis). While pathogens and microbiota co-exist, the interplay of them with host response remains enigmatic.

To date, the evolution of the animal intestine and microbiome is possibly the best described example of this interplay. For example, gut microbes not only metabolize plant tissues that ruminants cannot do by themselves but also protect them from pathogens and diseases. Microbiota transplantation has been used as a way of targeting microbiomes therapeutically against intractable diseases. Research is urged to depict the exact cellular and molecular mechanisms of this.

Recent advances in high-throughput, next generation sequencing and metabolomics have provided the tools to analyze microbial communities beyond the past census to defining community activities, especially in response to diet, host genetics, and other environmental factors. Tools are available to piece together how communities exclude, permit or control microbial pathogens or “put out the fire” of inflammation started by the pathogen and heal the breach in the mucosal layer. While the intrinsic complexity of microbiomes is a hurdle, research comprising the triad host-microbiota-pathogen is needed to understand the cause-effect relationships and mechanisms of disease; and what this emergent field needs most are studies that explain causation.

In this Research Topic, we present 4 timely articles that examined: perturbations to microbiome community structure in response to pathogens (Vargas et al.; Wu et al.; Yang et al.); community changes to severe disease (Yang et al.); amelioration of disease with specific community members (Vargas et al.); and identified community members possibly responsible for pathogen control (Li et al.). These studies looked at the microbiome of diverse animal species: termites (Wu et al.), zebrafish (Vargas et al.), chickens (Yang et al.) and bats (Li et al.) in response or association with fungal (Li et al.; Wu et al.) or bacterial pathogens (Vargas et al.; Yang et al.).

Yang et al. demonstrated a profound decline of ileal community diversity that correlated with the severity of necrotic enteritis in chickens. *Clostridium perfringens*, one of two agents needed to elicit this disease increased with disease severity. Other ileal members also increased in abundance (proteobacteria and enterococcal species), while there was a significant decline of *Lactobacillus* and Clostridial members. Changes in community structure also appeared to be tied to functional alterations in microbial metabolism of the chicken gut.

Vargas et al. looked at the protective effects of the fish microbiome, specifically two yeast strains (*Debaryomyces hansenii* and *Yarrowia lipolytica*) previously isolated from wild type zebrafish, against *Vibrio anguillarum* in conventional and germ-free zebrafish larvae. One yeast strain, *D. hansenii* 97 improved larvae survival. The microbiome alone improved survivability in larvae challenged with *V. anguillarum*. *Vibrio* infection stimulated neutrophil infiltration to the intestine, while yeast strains appeared to dampen this inflammatory response. While *V. anguillarum* affected intestinal community composition, pre-inoculation with either yeast strains prevented modification of 5 to 6 genera. Increase of the proteobacteria *Ensifer* and *Vogesella* appeared to be negative predictors of larval death in response to *V. anguillarum* infection.

Wu et al. found a profound decline in the termite hindgut microbiome in response to fungal infection with *Metarhizium robertsii*. There was discernible destruction of the bacterial endosymbionts in the fungus growing termite, *Odontotermes formosanus*, following exposure to *M. robertsii* spores. There was a decrease in diversity within the first 12 h of fungal infection, that was reflected in a decline with specific bacterial groups (Proteobacteria, Actinobacteria, and Gracilibacteria). However, there was a rebound in the intestinal microbiota to pre-infection levels.

Finally, Li et al. examined the bat's skin microbiota to elucidate how four bat species, present in China, are resistant to white-nose syndrome, despite the abundance of the fungal pathogen *Pseudogymnoascus destructans* in the cave environment. Several bacterial taxa were shared between the cave environment and the skin microbiota. There was notable variation in the abundance of bacterial taxa across cave sites, most notably *Pseudomonas, Rhodococcus*, and *Salinibacterium*. There was notable difference in gene relative abundances from KEGG orthology groups, most notably those associated with terpenoids and polyketides metabolism; functional categories involved in producing secondary metabolites, including anti-fungal products. These pathways present in the abundant taxa *Pseudomonas* and *Rhodococcus* may explain the resistance of these bat species to white-nose syndrome.

## Author Contributions

JM organized Research Topic, identified guest editors, served as editor, and wrote first draft of editorial. AS-B, TJ, and TC served as editors for this Research Topic, reviewed, and edited Research Topic description and editorial. All authors contributed to the article and approved the submitted version.

## Conflict of Interest

The authors declare that the research was conducted in the absence of any commercial or financial relationships that could be construed as a potential conflict of interest.

## Publisher's Note

All claims expressed in this article are solely those of the authors and do not necessarily represent those of their affiliated organizations, or those of the publisher, the editors and the reviewers. Any product that may be evaluated in this article, or claim that may be made by its manufacturer, is not guaranteed or endorsed by the publisher.

